# Exploration of Superspreading Events in 2015 MERS-CoV Outbreak in Korea by Branching Process Models

**DOI:** 10.3390/ijerph17176137

**Published:** 2020-08-24

**Authors:** Seoyun Choe, Hee-Sung Kim, Sunmi Lee

**Affiliations:** 1 Department of Mathematics, University of Central Florida, Orlando, FL 32816, USA; seoyunchoe@Knights.ucf.edu; 2 Department of Internal Medicine, Chungbuk National University Hospital, Chungbuk National University College of Medicine, Cheongju 28644, Korea; clint74@gmail.com; 3 Department of Applied Mathematics, Kyung Hee University, Yongin 17104, Korea

**Keywords:** MERS-CoV transmission dynamics, superspreading events, branching process models, control measures

## Abstract

South Korea has learned a valuable lesson from the Middle East respiratory syndrome (MERS) coronavirus outbreak in 2015. The 2015 MERS-CoV outbreak in Korea was the largest outbreak outside the Middle Eastern countries and was characterized as a nosocomial infection and a superspreading event. To assess the characteristics of a super spreading event, we specifically analyze the behaviors and epidemiological features of superspreaders. Furthermore, we employ a branching process model to understand a significantly high level of heterogeneity in generating secondary cases. The existing model of the branching process (Lloyd-Smith model) is used to incorporate individual heterogeneity into the model, and the key epidemiological components (the reproduction number and the dispersive parameter) are estimated through the empirical transmission tree of the MERS-CoV data. We also investigate the impact of control intervention strategies on the MERS-CoV dynamics of the Lloyd-Smith model. Our results highlight the roles of superspreaders in a high level of heterogeneity. This indicates that the conditions within hospitals as well as multiple hospital visits were the crucial factors for superspreading events of the 2015 MERS-CoV outbreak.

## 1. Introduction

The novel coronavirus emerged in December 2019 and spread to 214 countries with 16,897,243 confirmed cases and 663,470 fatalities as of 30 July 2020 [1]. South Korea was one of the countries that has experienced the early stage of the COVID-19 pandemic [2]. In the absence of vaccines and treatments, South Korea has implemented and maintained effective interventions such as large-scale epidemiological investigation, rapid diagnosis, social distancing, and prompt clinical classification of severe patients with appropriate medical measures. This was possible because the Korean government and health officials learned valuable lessons from the Middle East respiratory syndrome coronavirus (MERS-CoV) outbreak in 2015 [3].

The index case (the first infected individual) of the 2015 MERS-CoV outbreak in South Korea was a man who returned from a business trip in the Middle East. The man visited several medical clinics and hospitals because of fever (being infectious and unidentified), causing the rapid spread of MERS-CoV in the hospitals [3,4]. As a result, there were a total of 186 infected cases, including 38 deaths, and this event recorded the largest number of total confirmed cases outside the Arabian Peninsula.

The 2015 MERS-CoV outbreak in Korea is characterized as a nosocomial outbreak (infections occurred only in the hospital setting, not in the community) and by the dramatic inequality of transmission power, which led to many patients being infected by a small number of infected people. This event is called a “superspreading event” (SSE) and these infected people are called “superspreaders”. In general, these SSEs can be defined by a 20/80 rule, which means that ~80% of the effects come from 20% of the causes [5]. These SSEs were observed in outbreaks such as the 2003 severe acute respiratory syndrome (SARS) and Ebola outbreaks, many other infectious diseases [6,7]. To explore SSEs and superspreaders, there is a vast literature on using statistical analyses and mathematical models [8,9,10]. This research has revealed the individual heterogeneity and environmental variability of SSEs.

Mathematical modeling and simulations have been a useful tool for understanding the complex transmission dynamics of infectious diseases. Many studies have used mathematical models and statistical analysis for the 2015 MERS-CoV outbreak [11,12,13,14,15]. The stochastic models have been used the MERS-CoV outbreaks in the Arabian Peninsula [16,17]. They distinguished the transmission rates by zoonotic (index) cases and human-to-human secondary cases. The standard compartmental models have been used for the 2015 MERS-CoV by adding superspreading events to a standard SIR model [15,18]. The effects of isolation and control interventions were highlighted in the MERS-CoV dynamics. Agent-based models also were developed for the 2015 MERS-CoV outbreak [14,19]. These results underscored the importance of a loner-contact range of super-spreaders and corresponding interventions.

Another way of modeling SSE is a branching process model. In each discrete time step, an infected individual produces a random number of secondary infections that follow a certain probability distribution. There are a small number of cases whose value is far from the mean and these are used to model superspreaders. Pitchford et al. suggested three branching process models, each of whose random variable follows a different probability distribution [9]. Lloyd-Smith et al. developed branching process models to investigate the impact of individual variations on disease transmission dynamics [20]. A branching process model can incorporate the heterogeneity of individuals in terms of different probability distributions. Their results highlighted the roles of the SSEs in the 2003 SARS outbreaks and many other infectious diseases. Chowell et al. compared MERS-CoV and SARS to analyze the transmission characteristics and exposure patterns of SSEs [8] through a branching process model. Their study verified that the heterogeneity of MERS was higher than SARS, whereas the reproduction number of MERS was lower than SARS. For SARS, most of the infections occurred among healthcare workers, whereas MERS cases occurred among patients.

Superspreading events have been observed in the transmission dynamics of many infectious diseases. The 2015 MERS-CoV outbreak in South Korea has also shown superspreading events with a significantly high level of heterogeneity in generating secondary cases. It is critical to understand the mechanism for this high level of heterogeneity to develop effective intervention strategies and preventive plans for future emerging infectious diseases. In this regard, a branching process modeling approach is a useful tool for incorporating individual heterogeneity into the epidemic model. In this work, we employ a branching process model of the 2015 MERS-CoV transmission dynamics in South Korea. In particular, three different branching process models are employed to highlight the roles of superspreaders. The key components of the MERS-CoV outbreak have been estimated through the 2015 MERS-CoV epidemiological data. Furthermore, we explore the effects of three control measures, as it is valuable to understand the transmission dynamics of the 2015 MERS-CoV outbreak.

## 2. MERS-CoV Data

### 2.1. Data Sources

The 2015 MERS-CoV data in South Korea were publicly available from the Korea Centers for Disease Control and Prevention (KCDC), and the Ministry of Health & Welfare of South Korea [3]. Especially, KCDC disclosed information including epidemiological surveillance, hospitals, case contact tracing, and supersspreaders to the public [3]. Therefore, we gathered relevant information from the KCDC website, WHO, and news/media reports [3,4,21].

**Figure 1:**
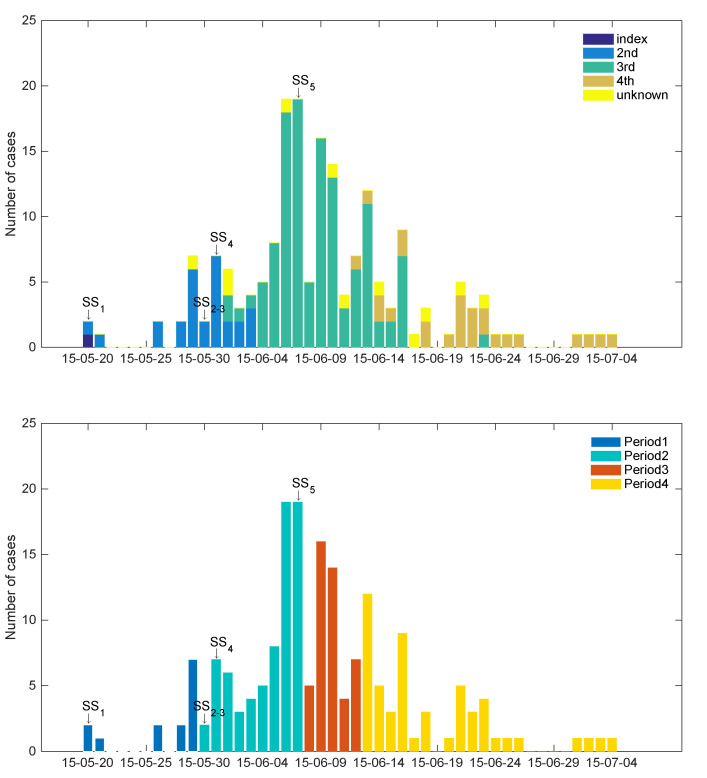
Time series of 2015 Middle East respiratory syndrome coronavirus (MERS-CoV) cases are displayed and classified by generations (**top**) and by intervention periods (**bottom**). The index case with 28 cases; the second generation with 111 cases; the third generation with 22 cases; the fourth generation with 1 case.

First, Figure 1 demonstrates the epidemic curves of the 2015 MERS-CoV outbreak according to generations (top panel) and intervention periods (bottom panel). There was a total of four generations, and we classified unidentified cases as unknown cases. A total of 28 secondary cases have been linked to the index patient in the first generation of the disease, 111 secondary cases have been reported for the second generation, 22 cases have been identified for the third generation, and one case has been reported for the fourth generation.

As seen in the bottom panel, we classified four periods according to interventions: period 1 (May 20–29) is the initial intervention period, during which we only included people who shared a room with the index patient or cared for the index patient; period 2 (May 30–June 7) is the second intervention period, during which we included further close contacts; period 3 (June 8–12) is the third intervention period after the government disclosed information regarding affected healthcare facilities on June 7; and period 4 (June 13–21) is the intervention period when the Republic of Korea and the World Health Organization (WHO) jointly announced the outbreak situation and stressed the awareness [4].

### 2.2. Superspreading Events

The 2015 MERS-CoV outbreak in Korea is characterized as a “superspreading event” (SSE). In general, these SSEs can be defined by a 20/80 rule, which means that 20% of infectious individuals are responsible for 80% of newly generated infections.

**Figure 2:**
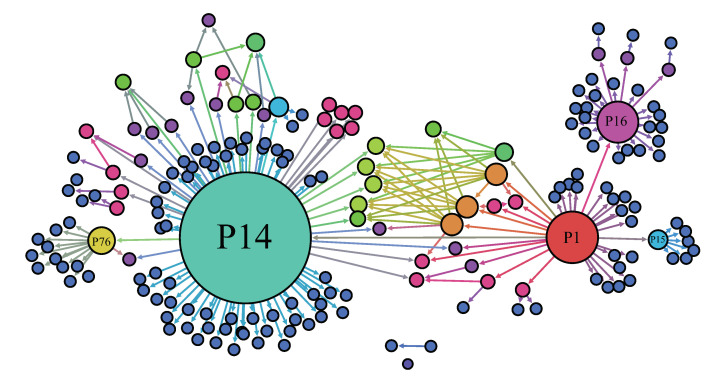
Transmission trees are displayed (the only infection tracing shown). The node size is proportional to the number of secondary infection cases (five superspreaders are identified).

**Figure 3:**
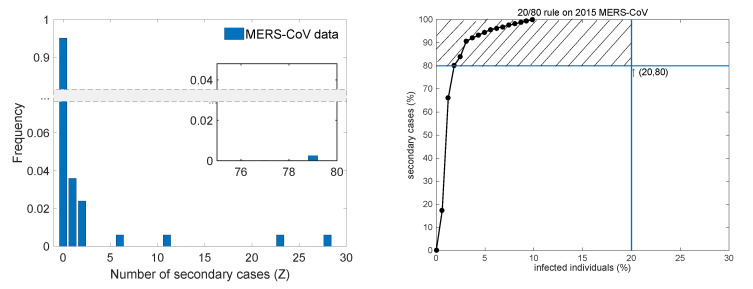
Distribution of the number of secondary cases (**left**) and the 20/80 rule of the 2015 MERS-CoV outbreak (**right**). In the right panel, an event that crosses the dashed area is called a superspreading event (SSE).

Transmission trees (infection tracing only) for a total of 186 MERS-CoV cases are displayed in Figure 2. The node size is proportional to the number of secondary cases (five superspreaders are identified). Figure 3 displays the distribution of the number of secondary cases (left panel) and the 20/80 rule (right panel) of the 2015 MERS-CoV outbreak. Distribution based on the data without multiple cases (17 patients who were infected by more than one patient) and two unknown cases. A total of 152 patients did not infect anyone, but one patient infected 79 people, indicating that the distribution shows high heterogeneity. Furthermore, it is called as an SSE when a cumulative percentage of secondary cases passes on the left of a (20,80) point, that is, the dashed area in the right panel [5]. In the case of the 2015 MERS-CoV outbreak, all infections originated from just 16 infected people of whom five were superspreaders. In other words, 79% (147 patients) of the total patients were infected by ~3% (five patients) of the patients. Table 1 lists the information about these superspreaders [3,22].

Table 1 displays the characteristics of these five superspreaders [3,22]. Note that although superspreaders #14 and #15 had similar conditions such as age, number of hospitals visited, and duration of exposure, there were enormous differences in the number of secondary infection; patient #14 infected 78 people in B Hospital and 1 person in X Hospital, and patient #15 infected only 6 people in C Hospital.

### 2.3. Nosocomial Infections

The 2015 MERS-CoV outbreak in Korea is characterized as a nosocomial outbreak (infections occurred only in the hospital setting, not in the community). The 2015 MERS-CoV outbreak mainly occurred in Seoul, Gyeonggi-do, and Daejeon. The index patient visited Hospital A and other hospitals and then had MERS infection confirmed in Hospital B. During his stay in Hospital A, the index case spread MERS to 26 patients, which included patients #14, #15, and #16. After contacted with the index case, #14 patient stayed in the emergency room of Hospital B and infected 78 people, and patient #15 infected six people in Hospital C. After patient #16, who was infected in Hospital A, went to Daejeon, he spread MERS to 13 and 10 patients in Hospitals D and E, respectively. After patient #76 came into contact with patient #14 in B Hospital, he spread MERS to five patients and four patients in Hospitals F and G, respectively.

**Figure 4:**
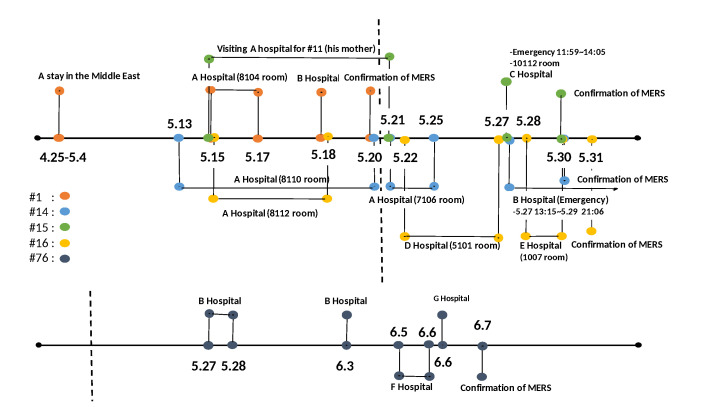
Multiple visits of the five superspreaders are displayed: the index case is shown with an orange circle, patient #14 is shown with a blue circle, patient #15 is shown with a green circle, patient #16 is shown with a yellow circle, and patient #76 is shown with a dark gray circle. The vertical dashed line indicates the date that the index case was confirmed on May 20.

There are studies in which multiple hospital visits are a key factor for superspreaders [22,23]. This is critical because visiting several hospitals increases the probability of contact and exposure with infectious individuals. Besides, there exists one more crucial factor of the hospital condition such as the density of emergency rooms, etc. Figure 4 displays the timeline and hospitals visited by the five superspreaders. The vertical dashed line shows the confirmed date of the index case, May 20 [24]. The orange circle indicates the index case and he came into contact with other patients during his stay in room 8014 in A Hospital. Patients #14, #15, and #16 are shown with blue, green, and yellow circles, respectively (upper line). Patient #76 who was infected in B Hospital is shown with a dark gray circle (bottom line).

As mentioned previously, patients #14 and #15 were different despite similar physical conditions. In comparison, after they left Hospital A, where they were exposed to the index case, patient #14 visited the emergency room of Hospital B and he stayed there for approximately 56 h, whereas patient #15 visited the emergency room of Hospital C for about 2 h and was moved to a ward (isolated) [25]. Furthermore, Hospital B, where patient #14 visited, had an emergency overcrowding index of over 100% [26]. Thus, the condition of hospitals and health-care facilities is also considered an important factor in the spread of 2015 MERS-CoV.

## 3. Branching Process Models

In this section, we consider the following branching process proposed in the previous work [20]. The random variable, *Z*, implies the number of secondary cases caused by each infectious individual. The offspring distribution of *Z* is modeled by a Poisson process. The value of ν for a given individual’s infectious history is the expected number of secondary cases they will cause, i.e., their individual reproductive number. Note that ν is an expectation and can take any positive real value, while *Z* is a non-negative integer (0,1,2,3,…). Three distinct scenarios of the individual reproductive number are considered, and therefore three candidate models for the offspring distribution are given as

- Branching process model 1 (BP1)
- Branching process model 2 (BP2)
- Lloyd-Smith (LS) branching process model

These models are classified by the distribution of individual reproductive number ν. BP1 is a generation- based model that neglects individual variation, which means that all individuals have the same reproductive number, $\nu =R_{0}$. Thus, the offspring distribution is $Z∼\mathrm{Poisson}(R_{0})$. BP2 assumes a homogeneous transmission rate with an exponentially distributed recovery rate as $\nu ∼\mathrm{Exponential}(1/R_{0})$. Then, the offspring distribution yields $Z∼\mathrm{Geometric}(R_{0})$. The LS model is a general formulation to incorporate models where ν is gamma-distributed with mean $R_{0}$ and a dispersion parameter *k*. The offspring distribution is $Z∼\mathrm{Negative}\;\mathrm{Binomial}(R_{0},k)$. Note that conventional notation is Z∼NegativeBinomial(p,k) where $p={\left(1+R_{0}/k\right)}^{-1}$.

When k→∞, $\mathrm{Negative}\;\mathrm{Binomial}(R_{0},k)$ becomes $Z∼\mathrm{Poisson}(R_{0})$, and also when k=1, it becomes $Z∼\mathrm{Geometric}(R_{0})$. In the negative binomial distribution, smaller values of *k* indicate greater heterogeneity in the secondary cases (see more details in [20]). In the negative binomial distribution of the LS model, we use the maximum likelihood method to estimate model parameters (two parameters). Further details can be found elsewhere [20,27,28].

A definition of a superspreader was proposed in [20], and the process uses a Poisson distribution with mean $R_{0}$ (the reproductive number) because a Poisson distribution means stochasticity without individual variation. Lloyd-Smith et al. defined an SSE as any infected individual who infects more than $Z^{\left(n\right)}$ others, where $Z^{\left(n\right)}$ is the *n*th percentile of the Poisson distribution with mean $R_{0}$. That is, in a homogeneous population, a 99th-percentile SSE means any case causing more infections than would occur in 99% of infectious histories. In the case of the 2015 MERS-CoV outbreak, there were five superspreaders, where the threshold number of cases $Z^{\left(99\right)}$ is 5. In the case of the 2015 MERS-CoV outbreak, we define superspreaders as those who infected five or more secondary cases according to the Poisson distribution. This is consistent with the definition of SSEs KCDC claimed (an infector with more than four infectees).

## 4. Numerical Results

### 4.1. Parameter Estimation

In this section, we estimate the parameters of the LS model using the maximum likelihood method. As mentioned earlier, heterogeneity in the MERS-CoV outbreak can be explained by both the reproduction number, $R_{0}$, and the dispersion parameter, *k*. The reproduction number represents the average number of secondary cases per index case. The dispersion parameter *k* quantifies the degree of heterogeneity in the secondary cases. Smaller values of *k* imply a higher level of heterogeneity in secondary cases.

**Figure 5:**
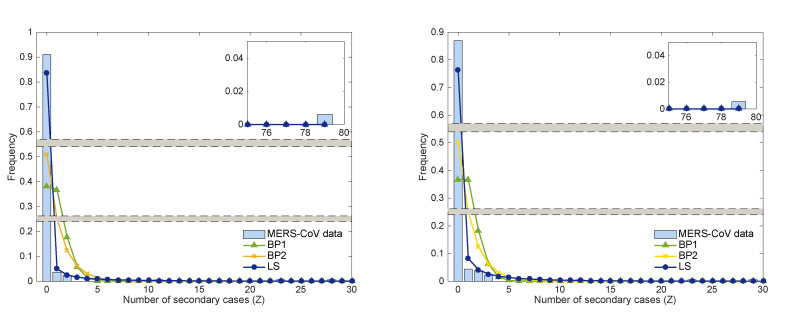
The distributions of secondary cases for the three models are compared with the MERS-CoV data; the **left** panel with k=0.063 and $R_{0}=0.96$ and the **right** panel with k=0.12 and $R_{0}=1.06$.

We have fitted a negative binomial distribution (the LS model) to the number of secondary cases from the empirical transmission tree of MERS-CoV data (as shown in Figure 2). Here, we consider two different scenarios depending on the information with/without multiple contacts in the secondary cases of 186 cases. The results of the estimated parameters are given in Table 2 under these two scenarios. The first one is the data set with a total of 167 cases (excluding 17 multiple contacts and 2 unknown) and the second is the data set with a total of 187 cases (including all cases). Figure 5 presents the results under these two scenarios.

Under the first set of 167 cases, the basic reproduction number ($R_{0}$ = 0.96) is below 1, while the basic reproduction number ($R_{0}$ = 1.06) is above for the second set of data with 186 cases. Moreover, the dispersion parameter is smaller, k=0.063 of the first data set (using 167 cases) than the one, k=0.12 of the second data set (using 186 cases). This shows that the parameters we obtained are sensitive to the transmission chains of the MERS-CoV outbreak.

Our estimation results of subcritical $R_{0}$ (which means that the basic reproduction number ($R_{0}$ = 0.96) is less than 1) are consistent with the other results in the previous work [8,16,29,30]. Their work also pointed out that the reproduction number for secondary cases during transmission chains of MERS-CoV in the Middle East has been estimated to lie below the epidemic threshold at $R_{0}=1$ [16,29,30]. Another study for the 2015 MERS-CoV in South Korea estimated $R_{0}=0.91$ and k=0.06 using the LS model [8].

Even though there are slight differences in the parameters estimated from the two sets of data, both results indicate that the heterogeneity of the secondary cases caused by an SSE is high. Furthermore, the extinction probability is the determination of the extinction of disease and our results have a large probability, 0.99 (or 0.98), which indicates that the outbreak should end eventually. It turned out that most MERS-CoV outbreaks have ended within a shorter period time.

**Figure 6:**
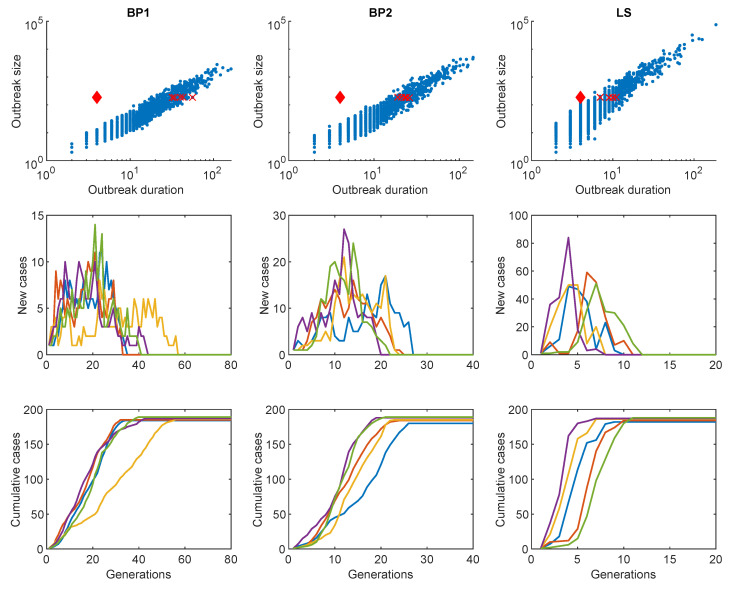
Scatter plot for the results of 5000 simulations on three models (upper panels); the red diamond mark indicates the cases of the 2015 MERS-CoV outbreak (fourth generation; outbreak size, 186 cases). The middle panels show the results of five incidence cases that are the nearest cases to the 2015 MERS-CoV outbreak according to each model (red cross marks in each scatter plot) and the bottom panels show the cumulative cases of the five incidence cases.

Next, we illustrate the results of MERS-CoV incidence using the three branching process model given in the previous section. The parameters of the LS model will be used for the BP1 and BP2 models so that they have the same $R_{0}$. Figure 6 shows the results of 5000 simulations with the parameters (k=0.063 and $R_{0}=0.96$). As seen in the upper panels, the LS model has a greater scope for outbreak size and outbreak duration than BP1 and BP2. This is because of the greater probability of a large outbreak size in the LS model, which has high heterogeneity. The red diamond mark indicates the 2015 MERS-CoV outbreak, which is a 4th generation outbreak with a total outbreak size of 186 cases. This again confirms that the results of the LS model capture the actual 2015 MERS-CoV outbreak the best.

The middle panels of Figure 6 show the five outbreaks that are the nearest cases to the 2015 MERS-CoV outbreak for each model (five red cross marks) and the bottom panels are the cumulative cases for the outbreaks. This demonstrates that the LS model has the largest peak size and the shortest outbreak duration due to the highest level of heterogeneity (or superspreading events). This implies there is more chance to have a rapid increase in the outbreak size within a short time window (smaller generations). These epidemic outputs of the three models are compared in Table 3. Although the extinction probabilities of BP1 and LS are slightly below 1, they are almost 1 because of $R_{0}<1$ and this is consistent with the previous research [8]. This confirms that the MERS-CoV showed a significantly high level of heterogeneity in secondary cases due to the five superspreaders.

**Figure 7:**
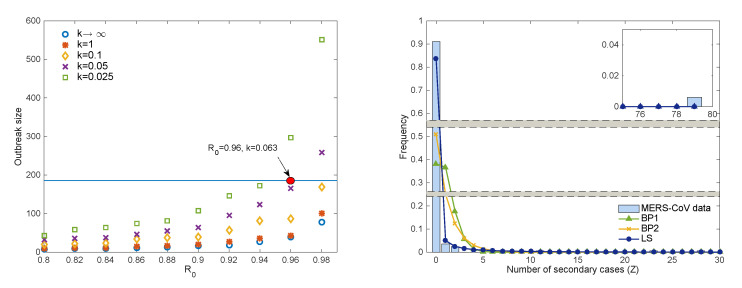
Outbreak size for several $R_{0}$ and *k*. Each result is the mean of 5000 simulations. The blue line indicates 186 patients of MERS-CoV in South Korea (left), 2015 MERS-CoV data (bar), and the results of the three models with parameters of best-fitting (red filled circle; k=0.063 and $R_{0}=0.96$ with 167 cases).

We present the impact of the basic reproduction number, $R_{0}$, and the dispersion parameter *k* on the outbreak size. The left panel of Figure 7 demonstrates the averaged outbreak size as varying $R_{0}$ and *k* values. The blue line indicates a total of 186 cases of the 2015 MERS-CoV. The result shows that the outbreak size increases as the dispersion parameter *k* decreases. Besides, the outbreak size is highly influenced by *k* as $R_{0}$ increases. When $R_{0}=0.96$ and k=0.063 (red filled circle), the result is the closest to the actual 2015 MERS-CoV outbreak.

The right panel of Figure 7 illustrates the results of three models using $R_{0}=0.96$ and k=0.063 of the LS model. Blue bars show the frequency of secondary cases of the 2015 MERS-CoV outbreak compared with the results of the three models: BP1 is a green triangle curve, BP2 is a yellow cross curve, and LS is a blue circle curve. The probability of no secondary infection Pr(Z=0) is compared using the three models: Pr(Z=0) = 0.38 (BP1), Pr(Z=0) = 0.51 (BP2), and Pr(Z=0) = 0.85 (LS), respectively. The LS model captures the secondary cases in the 2015 MERS-CoV outbreak, indicating that the 2015 MERS-CoV outbreak had enormous heterogeneity.

### 4.2. The Effect of Control Measures

We investigate the effects of three control measures on the MERS-CoV transmission dynamics. These control measures are proposed in the work [20]. Population-wide control is where infectiousness of individual is totally reduced by a factor *c* so the individual reproduction number is reduced $\nu_{c}^{pop}=\left(1-c\right)\nu$, that is, $Z_{c}^{pop}∼\mathrm{NegB}\left(\left(1-c\right)R_{0},k\right)$. Individual-specific control is where $Z_{c}^{ind}=0$ as a factor *c* if controlled, and $Z_{c}^{ind}=Z$ if not controlled. Furthermore, in individual-specific control, random individual-specific control is where individuals are randomly controlled, and targeting individual-specific control is where the top 20% exercise more control effort than the bottom 80% (in our simulations, the top 20% expend four times more effort than the bottom 80%). Targeting individual-specific control is particularly for controlling superspreaders (the top 20%).

**Figure 8:**
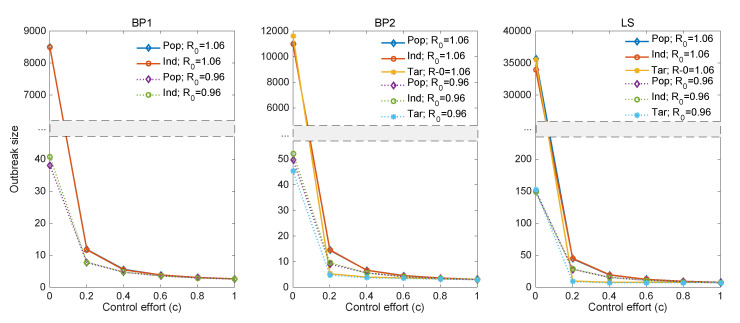
The impacts of control efforts on the outbreak size under the three models: solid lines indicate higher $R_{0}$ and dashed lines indicate lower $R_{0}$. Population control is shown with blue and purple lines, random individual control is shown with red and green lines, and targeting individual control is shown with yellow and magenta lines.

**Figure 9:**
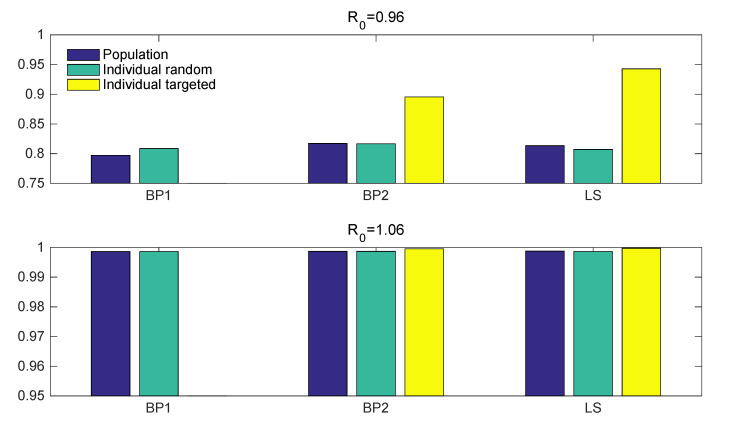
The reduction rate of the three models when the control effort is 20%. The upper panel is for $R_{0}=0.96$ and the bottom panel is for $R_{0}=1.06$. The blue bars denote population control, green bars denote random individual control, and the yellow bars denote targeting individual control.

**Figure 10:**
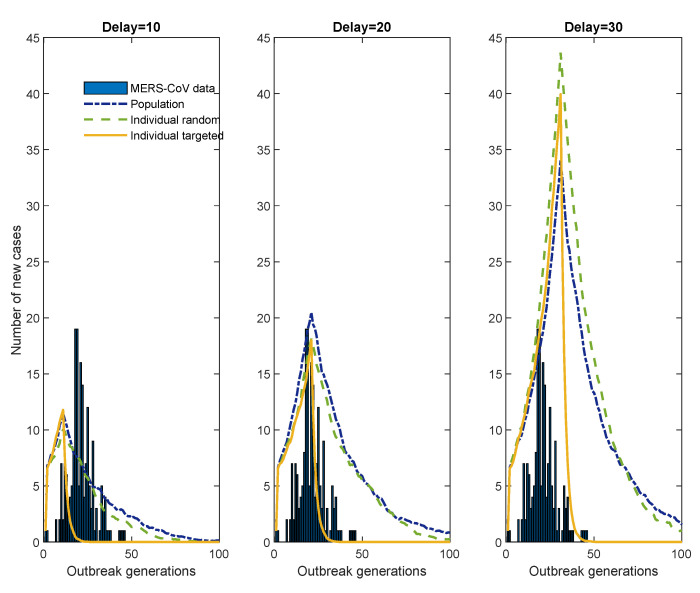
The impact of the timing of starting control measures: the blue bar graph shows the epidemic curve of 2015 MERS-CoV, blue dash-dotted lines denote population control, green dashed lines denote random individual control, and yellow solid lines denote targeting individual control.

Figure 8, Figure 9 and Figure 10 illustrate the results of control strategies for the three models: population-wide control, random individual-specific control, and targeting individual-specific control. To compare the results of different $R_{0}$, this simulation used two $R_{0}$ values ($R_{0}=0.96$, $R_{0}=1.06$) and k=0.06; all the results were the average of 10,000 simulations and the total outbreak size of 100 generations. In all simulations, BP1 did not have targeting individual-specific control because all individuals are identical in BP1, which means that random individual-specific control and targeting individual-specific control are the same.

The left panel of Figure 8 shows the results of BP1; the results of population-wide control and random individual-specific control are similar. For BP2 (the middle panel) and the LS model (the right panel), although there is a slight difference between models without control (c=0), the outbreak size of targeting individual-specific control was lower than those of other controls (yellow solid line $R_{0}=1.06$ and magenta dotted line $R_{0}=0.96$). In particular, despite the larger outbreak size of higher $R_{0}$ (=1.06), the outbreak sizes of two $R_{0}$ values have no significant difference after a control effort of 0.2. Furthermore, the reduction rates for all models remain similar after the control effort reaches 0.2.

Next, Figure 9 illustrates the reduction rates of the three models when the control effort is 0.2. Although there is no significant difference between reduction rates of control measures for BP1 when $R_{0}=0.96$, the reduction rates of targeting individual-specific controls (yellow bars) for BP2 and LS are 89% and 94%, respectively, which indicates that targeting control is more effective than random individual-specific controls (green bars) and population-wide controls (blue bars). The results show that the reduction rate of all models is over 75% when $R_{0}$ is 0.96 and the reduction rate of all models is over 99% when $R_{0}$ is 1.06. Accordingly, targeting control is the most effective control measure even with a 20% control effort.

Figure 10 presents the results of the LS model only with $R_{0}=1.06$, k=0.06, and the control effort level c=10%. The timing of starting control measures is varied as 10, 20, or 30 days delay. The bar graph displays the 2015 MERS-CoV data, the blue dash-dotted line denotes population control, the green dashed line denotes individual-random control, and the yellow solid line denotes individual-targeting control in each panel. As shown in the middle panel, when the control delay is 20 days, the outbreak size with population control is 700, with the individual-random control it is 619, and with the individual-targeting control it is 252. Note that the outbreak size with individual-targeting control increases to 631 when the control delay is 30 days, whereas the outbreak size is 111 when the control delay is 10 days. These results highlight the timing of control efforts combined with the control level are critical factors to reduce the outbreak size greatly.

## 5. Discussion

We have clarified the characteristics of an SSE and superspreaders during the 2015 MERS-CoV outbreak in Korea. Our study suggests that although visiting multiple hospitals plays a key role in the characteristics of superspreaders, the condition of hospitals may also be associated with the number of superspreaders and the 2015 MERS-CoV outbreak. Specifically, we compared patients #14 and #15 and found that despite similar conditions, the significant difference in the number of infections (79 vs. 6) may be due to the exposure time they stayed in the emergency room (56 h vs. 2 h).

We also analyzed the 2015 MERS-CoV outbreak with a branching process and presented the effect of control measurements. The LS model provides a better fit than the other models (BP1 and BP2). The values of the dispersion parameter *k* in the LS model are very small, which means that the infectiousness of individuals in the 2015 MERS-CoV outbreak in Korea shows a significant heterogeneity, representing clear evidence of it being an SSE. Furthermore, the extinction probability has almost reached 1 and this suggests that the 2015 MERS-CoV outbreak in South Korea would soon disappear. The results of control measures indicate that targeting individual-specific control is the most effective and even just 20% control effort for all models is effective. Moreover, when the delay time is 20, it best describes the 2015 MERS-CoV outbreak, and the disclosure of hospital lists occurred about 20 days after the index patient was confirmed. This result suggests that if the implementation of the Korean government had been later, the 2015 MERS-CoV outbreak would have been about twice as large.

This research has several limitations. First, multiple-contact cases that were infected by several patients were excluded for clarity. Our findings may be different if the dataset included all multiple-contact cases. However, although 19 cases were not included, it may be better to use such partial data than data that is ambiguous and unclear. Second, a spatial structure has not been incorporated into our model. This can be resolved in our future research by using a network structure model. Despite these limitations, our study is valuable in that we explore other characteristics of superspreaders and control measures of the branching process model on the 2015 MERS-CoV in South Korea. Our results highlight the roles of superspreaders in a high level of heterogeneity. This indicates that the conditions within hospitals as well as multiple hospital visits were the crucial factors for superspreading events with a high level of heterogeneity of the 2015 MERS-CoV outbreak. Therefore, public health officials should take accounts of these factors into future intervention strategies of emerging infectious diseases. 

## Figures and Tables

**Table 1:** Characteristics of the five superspreaders.

	# 1	# 14	# 15	# 16	# 76
Age (years)	68	35	35	41	75
Gender	Male	Male	Male	Male	Female
Number of secondary infections	28	79	6	23	11
Number of contacts	626	594	304	277	805
Exposed hospitals	—	A	A	A	B
Number of hospitals visited	5	3	3	4	3
Duration of exposure	April 29–May 2	May 15–17	May 15–17	May 15–17	May 27–28
Underlying disease	Hypertension	—	Liver failure	Pancreatitis	Multiple myeloma

**Table 2:** Parameter estimation results of the LS model; estimates of the reproduction number $R_{0}$ and the dispersion parameter *k* of the 2015 Middle Eastern respiratory syndrome (MERS-CoV).

Parameter	*N*	Mean $R_{0}$ (95% CI)	*k* (95% CI)	Pr (Extinct)
Value	167	0.96 (0.6915, 1.2285)	0.063 (0.0451, 0.0809)	0.99
Value	186	1.0 (0.7720, 1.3480)	0.12 (0.1008, 0.1392)	0.98

**Table 3:** Simulation results for the three models ($R_{0}=0.96$ and k=0.063 ) are compared with the MERS-CoV data.

	BP1	BP2	LS	MERS-CoV Data
Outbreak size	41.19	59.22	140.06	186
Generations	8.47	7.25	4.37	4
Number of secondary infections of the index case	12	14	102	79
Peak size	4.06	5.77	17.13	19
Peak generation	4.41	4.02	2.67	3
Extinction probability	0.99	1	0.99	—

## Abbreviations

The following abbreviations are used in this manuscript.

BP	Branching process
LS	Lloyd-Smith et al.
MERS-CoV	Middle East respiratory syndrome coronavirus
SARS	Severe acute respiratory syndrome
SSE	Superspreading event
